# Biomimetic cell-actuated artificial muscle with nanofibrous bundles

**DOI:** 10.1038/s41378-021-00280-z

**Published:** 2021-09-03

**Authors:** Yongwoo Jang, Sung Min Kim, Eunyoung Kim, Dong Yeop Lee, Tong Mook Kang, Seon Jeong Kim

**Affiliations:** 1 Center for Self-Powered Actuation, Department of Biomedical Engineering, Hanyang University, Seoul, 04763 South Korea; 2 Department of Physical Education and Human-Tech Convergence Program (BK21 Four), Hanyang University, Seoul, 04763 South Korea; 3 Department of Physiology, Sungkyunkwan University School of Medicine, Suwon, 16419 South Korea

**Keywords:** Nanofabrication and nanopatterning, Carbon nanotubes and fullerenes

## Abstract

Biohybrid artificial muscle produced by integrating living muscle cells and their scaffolds with free movement in vivo is promising for advanced biomedical applications, including cell-based microrobotic systems and therapeutic drug delivery systems. Herein, we provide a biohybrid artificial muscle constructed by integrating living muscle cells and their scaffolds, inspired by bundled myofilaments in skeletal muscle. First, a bundled biohybrid artificial muscle was fabricated by the integration of skeletal muscle cells and hydrophilic polyurethane (HPU)/carbon nanotube (CNT) nanofibers into a fiber shape similar to that of natural skeletal muscle. The HPU/CNT nanofibers provided a stretchable basic backbone of the 3-dimensional fiber structure, which is similar to actin-myosin scaffolds. The incorporated skeletal muscle fibers contribute to the actuation of biohybrid artificial muscle. In fact, electrical field stimulation reversibly leads to the contraction of biohybrid artificial muscle. Therefore, the current development of cell-actuated artificial muscle provides great potential for energy delivery systems as actuators for implantable medibot movement and drug delivery systems. Moreover, the innervation of the biohybrid artificial muscle with motor neurons is of great interest for human-machine interfaces.

## Introduction

In most mammals, muscle fibers are the individual contractile units that are organized into individual bundles of elongated multinuclear myocytes^1,2^. The bundled myofilaments are bound together by connective tissue, which is a unique structure that regulates the force and mechanical strength of muscle tissue^1,2^. Various attempts to mimic the function and/or structure of skeletal muscle have been introduced and applied to artificial muscle using biocompatible and functional materials^3–5^. To further facilitate biological integration into artificial muscle, this study developed a biohybrid artificial muscle of living muscle fiber and scaffolds inspired by skeletal muscle.

In an artificial muscle, a fiber-shaped structure is useful in creating higher-order assemblies by bundling, weaving, and folding^6^. In addition, anatomical and structural studies on skeletal muscle tissue support the importance of fibrous bundles in muscular functions, such as the force regulation and mechanical strength of muscle tissue^7^. Hence, fiber-shaped structures have been created for muscle tissue regeneration using cellular constructs and for artificial muscle using various functional materials^4,8–10^.

The integration of living muscle cells into soft materials is promising for soft robots due to their actuation^11–16^. Over the past few decades, there has been considerable progress in the development of biohybrid actuators actuated by muscle cells, such as jellyfish and rays^11–16^. Electrical or optical control enables biohybrid actuators to move through the contractions of integrated muscle cells^11–16^. On the other hand, artificial muscles have been developed to mimic physiological muscle for various actuators^17,18^. Most studies in artificial muscle have employed multifarious functional materials, including hydrogels, nylon, and CNTs^3–5,19^. As shown in biohybrid actuators, an advanced concept of biohybrid artificial muscle has been recently suggested to integrate skeletal muscle cells on CNT sheets^20^. However, the biohybrid muscle is still limited to a two-dimensional (2D) matrix. Therefore, we introduce a biohybrid artificial muscle with a nanofibrous bundle, as observed in natural skeletal muscle.

To enhance the alignment and performance of hybrid artificial muscle, we hybridized fibrously differentiated skeletal muscle cells and nanofibrous scaffolds. In more detail, we fabricated nanofibers of hydrophilic polyurethane (HPU) using the electrospinning method and overlapped nanofiber CNT membranes to improve the flexibility and mechanical strength. Furthermore, we integrated skeletal muscle cells into the HPU/CNT matrix and fabricated fiber bundles using the biscrolling method^21^. Consequently, the cell-laden hybrid artificial muscle was a bundled fiber-containing nanofibers of HPU, CNTs, and elongated skeletal cells that demonstrated contractive behaviors in response to an applied electrical stimulation. Therefore, cell-actuated biohybrid artificial muscle has great potential for use in cell-based robotic systems and drug delivery systems.

## Results and discussion

**Fig. 1:**
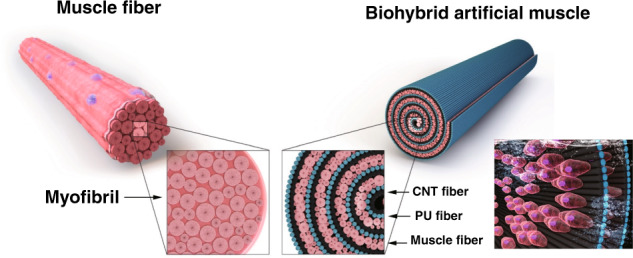
Schematic illustration of biohybrid artificial muscle. Muscle fiber consists of a bundle of myofibril (left). Similar to the muscle fiber, biohybrid artificial muscle is composed of CNT fiber, PU fiber, and skeletal muscle fiber (right)

A cell-actuated biohybrid artificial muscle is illustrated in Fig. 1. For biohybrid artificial muscle, we first attempted to combine nanofibers, hydrophilic polyurethane (HPU), and carbon nanotubes (CNTs) to achieve a strong and flexible scaffold. Among various polyurethane polymers, we selected HPU because hydrophilicity is an advantageous condition for skeletal cells and fabricated nanofibers using the electrospinning method. Figure S1A shows highly aligned HPU nanofibers on the collector. The electrospun HPU nanofibers were attached to the cover slide and then overlapped with the CNT sheets drawn from a nanotube forest to improve the mechanical strength, as shown in the SEM image of Figure S1B. The HPU/CNT matrix exhibited a full strain reversibility of up to 4% in the stretching and releasing cycle (Fig. S2A) and a 3.55 (±0.41) electrical conductance (Fig. S2B).

**Fig. 2:**
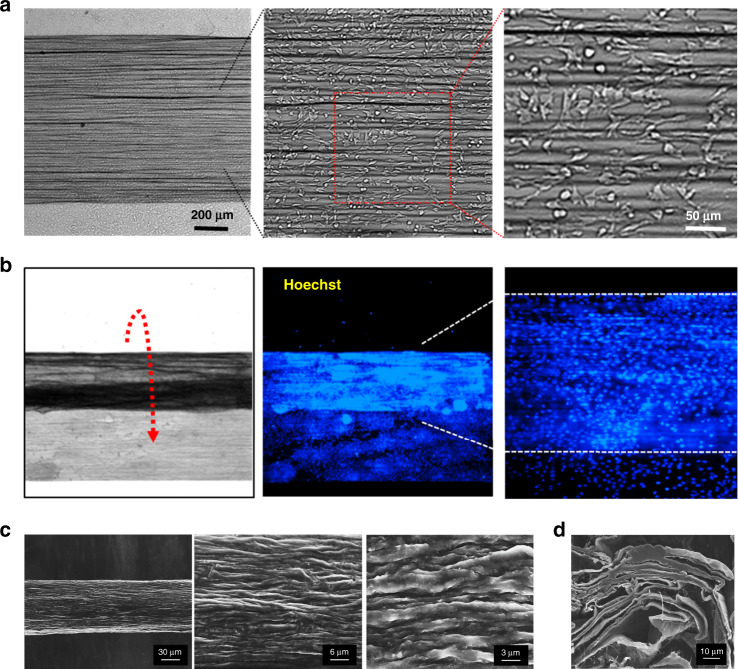
The fabrication of biohybrid artificial muscle. **a** C2C12 cells on collagen-coated PU/CNT matrix. **b** 3D fiber structure fabrication using a biscrolling C2C12 cell-laden PU/CNT matrix. Blue fluorescence indicates each cell nucleus stained with Hoechst dye. **c** SEM image of biscrolled C2C12 cell-laden PU/CNT fiber. **d** Cross-sectional SEM image of biscrolled C2C12 cell-laden PU/CNT fiber

To facilitate the differentiation of C212 myoblasts, a nanofibrous HPU/CNT matrix was uniformly coated with collagen (4 mg mL^−1^), which is a main extracellular component for maintaining skeletal muscle cells^22^. Thereafter, C2C12 myoblast cells (5 × 10^5^ mL^−1^) were seeded on collagen-coated HPU/CNT planar matrix and further maintained in a CO_2_ incubator for 5 h to allow attachment time on the matrix. Consequently, the scattered myoblasts anchored and spread out on the collagen-coated HPU/CNT scaffold (Fig. 2a). The attached myoblasts continuously proliferated, isotropically aligning along the CNT scaffold in the growth medium (Fig. S3). In fact, nanofibrous scaffolds are frequently used to align the incorporated cells for tissue engineering^23–25^.

To incorporate living myoblasts into the 3D microstructure, we attempted to biscroll the cell-containing HPU/CNT matrix in a parallel direction to a row of CNT nanofibers 5 h after seeding (Fig. 2b, optical image). The aligned CNT nanofibers served as the basic backbone of the 3D fiber structure, which is similar to actin-myosin structural scaffolds in muscle tissue. In addition, the viscosity of hydrophilic PU containing water enabled an adhesive to maintain the biscrolled structure. To visualize scrolled cells, we stained the myoblast cells with fluorescent Hoechst dye, which incorporates into the cell nucleus. The fluorescent image indicated that myoblasts were isotropically embedded along the inside of the scrolling HPU/CNT matrix. Therefore, the biscrolled fiber could structurally load many cells per unit area and had the advantage of arranging HPU/CNT nanofibers in one direction.

The SEM image of Fig. 2c presents a final biscrolled cell-laden HPU/CNT fiber. The higher magnification SEM images provide a porous surface composed of nanofibers in the biscrolled fiber, which is likely to facilitate the delivery of nutrients and oxygen to skeletal cells. The cross-sectional SEM image shows a layer-by-layer structure in the biscrolled C2C12 cell-laden PU/CNT fiber (Fig. 2d).

**Fig. 3:**
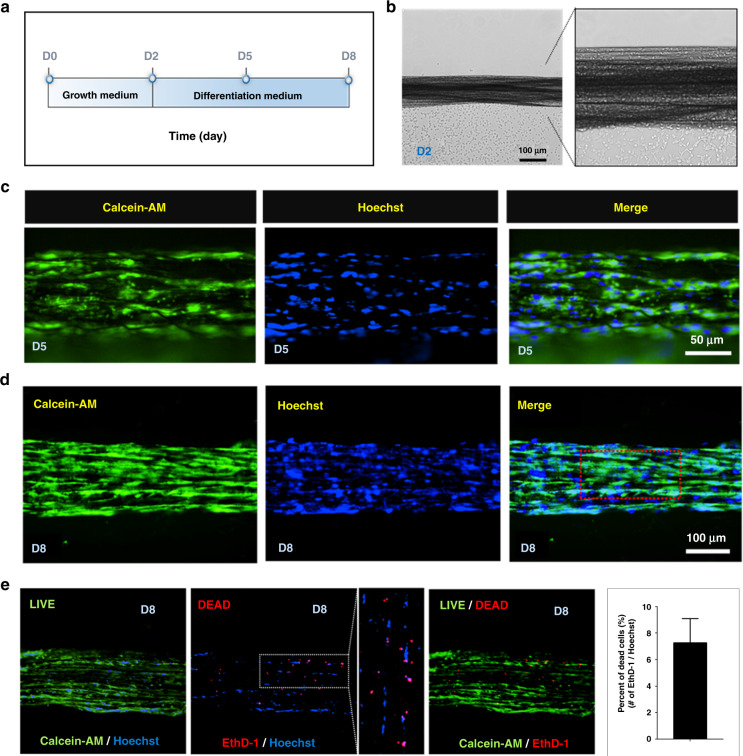
Morphology and viability of incorporated myoblast cells. **a** Schematic representation of myogenic differentiation of C2C12 cells in the biscrolled fiber. The cell-laden fibers were maintained in growth medium for 2 days and subsequently incubated in the differentiation medium for 6 days. **b** Optical image of cell-laden fiber at day 2 (D2) prior to the change to the differentiation medium. **c** Fluorescence photomicrograph of cell-laden fibers at day 5. Calcein-AM (green) and Hoechst (blue) dyes were used to stain the cytosolic compartment and nuclei of the cells, respectively. Dual fluorescence shows multinucleated myocytes. **d** Fluorescence photomicrograph of cell-laden fibers at day 8. Individual bundles of elongated multinuclear myocytes were observed along the fiber. **e** Live/dead images of the cell-laden fibers at day 8. The bar graph indicates the percent of dead cells, which are defined as the number of EthD-1-positive cells divided by total cells

As shown in Fig. 3a, we induced the proliferation (for 2 days) and differentiation (for 6 days) of incorporated myoblast cells for functional muscle fiber after biscrolling. For proliferation, the cell-laden fiber was incubated in the growth medium for 2 days to increase the number of incorporated cells (Fig. 3b). Subsequently, we maintained the cell-laden fiber in the differentiation medium for 6 days to induce the myotube fusion of myoblasts into multinucleated fibers. To characterize the multinucleated muscle fibers, we attempted to label the cell nucleus and cytosolic compartment at day 5 using two fluorescent dyes, Hoechst and calcein-AM, for the nucleic and cytosolic compartments, respectively. Consequently, the merged fluorescent image supports the presence of multinucleated myoblasts during differentiation (Fig. 3c).

At day 8, we finally assessed the cell-laden fiber using the two fluorescent dyes mentioned above. Green fluorescence was strongly observed in each array of discrete multinucleated myotubes, which is a unique phenotype of differentiated skeletal muscle cells, indicating that incorporated cells successfully survived by forming nanofibers similar to physiological muscle fibers (Fig. 3d). In addition, we assessed cell viability, revealing a proportion of live and dead cells of embedded myoblasts in biohybrid artificial muscle. Polyanionic calcein-AM is retained within live cells (green fluorescence), and ethidium homodimer-1 (EthD-1) is incorporated into the nucleic acids of dead cells with damaged membranes (red fluorescence), as shown in Fig. 3e. Calcein-positive live cells were observed in almost all regions of the biohybrid muscle, whereas EthD-1-positive dead cells were observed in scattered parts of it, indicating ~7% dead cells (7.27 ± 0.82) (Fig. 3d). Taken together, the myoblasts incorporated into HPU/CNT fibers were structurally differentiated into myotubes along hybrid scaffolds without a large population of cell death.

**Fig. 4:**
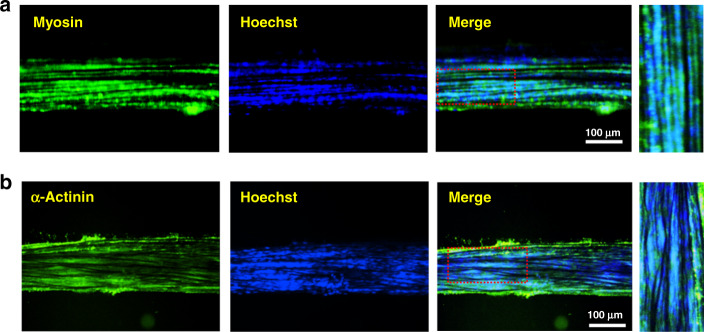
The terminal differentiation of incorporated myoblast cells. Immunostaining of hybrid artificial muscle with anti-myosin (**a**) and anti-α-actinin (**b**), which are myogenic markers expressed in terminally differentiated muscle fibers

To further investigate functional differentiation into skeletal muscle, biohybrid artificial muscle was stained for myogenic markers, such as myosin and α-actinin, which are expressed in differentiated muscle fibers. Immunocytochemical analysis revealed that Hoechst-positive cells were costained with myosin (Fig. 4a) and α-actinin (Fig. 4b). These results demonstrated that the embedded myoblasts were structurally transformed from HPU/CNT fibers into functional muscle fibers.

**Fig. 5:**
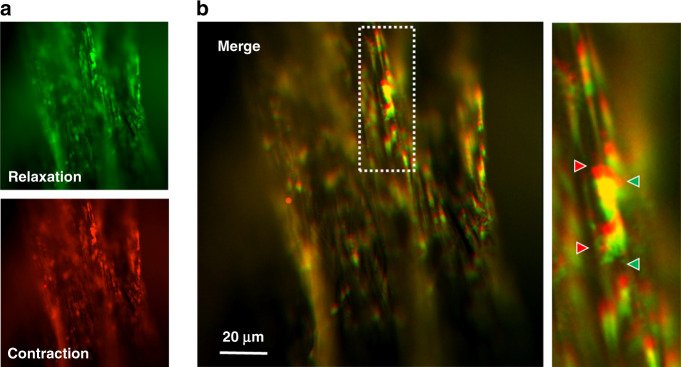
Contraction behaviors of biohybrid artificial muscle. **a** Fluorescence photomicrograph of the relaxation state (green) and contraction state (red) of biohybrid artificial muscle in response to an applied electrical field. **b** Merged image of relaxation and contraction states. The distance between the red arrow and green arrow indicates the dislocated distance of the biohybrid artificial muscle

To test the contractile activity of biohybrid artificial muscle, we first labeled a fluorescent dye to easily trace the actuation and stimulated an electric field to excite muscle fibers at a frequency of 0.5 Hz with a 15-ms step pulse duration (80 V). A schematic diagram and real picture of the device with a detection system and electric field stimulation for tracing contraction and relaxation are shown in Fig. S4. Interestingly, the biohybrid artificial muscle contracted and was reversely released upon electric stimulation, similar to the behavior of natural skeletal muscle (Supplementary Video 1). Figure 5a shows representative images of relaxation (green) and contraction (red) of a biohybrid artificial muscle. The merged image of relaxation (green) and contraction (red) is presented in Fig. 5b. The inset image indicates the dislocated distance (~2.73 ± 0.27 μm) of the biohybrid artificial muscle. According to previous studies, electrical pulse stimulation (1–2 Hz) of differentiated C2C12 cells shows an ~2–4 μm contractile distance, which was evaluated as the distance shortened between specified points^26–28^. In this study, differentiated C2C12 myotubes on PU/CNT scaffolds seemed to exhibit maximum contractile activity by electrical pulse stimulation. It is thought that the cell-incorporated fiber will be able to improve contractile movement by the deformation of structures such as coils and helices^19,29^. In terms of durability, theoretically, incorporated cells can be used semipermanently once they are nourished. However, further studies are warranted to clarify the durability of our biohybrid artificial muscle in terms of how long this contractile activity can be maintained in both in vitro and in vivo environments.

### Conclusions

In conclusion, this study shows a biohybrid artificial muscle fabricated from skeletal muscle cells and PU/CNT scaffolds, inspired by skeletal muscle tissue. The HPU nanofiber made the biohybrid muscle hydrophilic and stretchable in aqueous conditions, improving the attachment and differentiation of skeletal cells. In addition, nanofibrous CNTs further provided electrical conduction and mechanical strength to the biohybrid muscle. The scattered C2C12 myoblast cells on the nanofibrous HPU/CNT planar matrix were biscrolled into a fiber shape and further induced proliferation and differentiation. Consequently, multinucleated myoblast cells were aligned in the interspaces along nanofiber HPU/CNT scaffolds, resulting in a multifibrous bundle with similar morphology to that of physiological skeletal muscle fibers. In hybrid artificial muscle, a noticeable advantage of native cell integration is to remotely control the integrated cells through genetic engineering. For example, skeletal muscle cells that overexpress optogenetic genes, such as channel rhodopsin and halorhodopsin, can respond to a specific wavelength of light^16,30^. Therefore, the further application of genetically modified skeletal cells to biohybrid artificial muscle seems likely to expand their functional applications in emerging cell-based soft robotic systems. In addition, since the biohybrid artificial muscle consists of skeletal muscle, it can be controlled by the motor nerve once motor neurons are innervated, and it is also controlled by bimolecular acetylcholine, a neurotransmitter that regulates the contraction of muscle similarly to physiological muscle tissue. Therefore, it is also of great interest for a human-machine interface to innervate the biohybrid artificial muscle with motor neurons.

## Materials and methods

### HPU electrospinning

HPU nanofibers were electrospun from a 5 wt% solution of HPU in 95% ethanol. Using a syringe pump, 5 wt% HPU solution in a stainless-steel needle was fed to the parallel metal collectors. A voltage of 15 kV was applied between a syringe needle and parallel metal collectors using high-voltage DC power supplies (MHP40-02A, model IHPS 40 kV, 2 mA, Wookyung Tech). The collected HPU nanofiber was moved onto a cover glass.

### HPU/CNT matrix fabrication

The CNT sheets were drawn from a CNT forest fabricated by a chemical vapor deposition method. A layer of CNT sheet overlapped on the HPU nanofibers attached to the cover glass.

### Myoblast culture

Myoblast C2C12 cells were maintained in DMEM supplemented with 10% fetal bovine serum (FBS) under the previously reported conditions^31^. As shown in Fig. S5, we confirmed tube formation, which is a unique feature of skeletal differentiation, in DMEM supplemented with 2% horse serum for 6 days. To facilitate myogenic differentiation, we further supplemented insulin-like growth factor-1 (IGF-1) during differentiation, in accordance with previous reports^32,33^. This process significantly increased the myogenic index, which is defined as the number of cells containing three or more nuclei divided by the total number of nuclei in Hoechst-positive cells (Fig. S6). For the experiment, myoblast C2C12 cells were cultured on a collagen-coated HPU/CNT nanofiber scaffold. Attached C2C12 cells were incubated in growth medium with 10% FBS for 2 days and then induced to differentiate in DMEM supplemented with 2% horse serum and IGF-1 (50 ng mL^−1^) for 6 days.

### Immunocytochemistry

Immunostaining of the cell-laden fiber was performed to evaluate the terminal differentiation of C2C12 cells, as previously described^34^. The cell-laden fibers were placed in 4% paraformaldehyde for 30 min to fix the incorporated cells. Primary antibodies raised against skeletal muscle myosin (Santa Cruz, sc-32732) or α-actinin (Abcam, ab9465) were incubated overnight at 4 °C on a rotary shaker. One day later, the cell-laden fiber was washed thrice with phosphate buffer solution containing 0.5% Triton X-100 and subsequently incubated for 1 h at room temperature with Alexa Fluor 488-conjugated anti-mouse IgG (Invitrogen) and Hoechst 33342 (Invitrogen).

### LIVE/DEAD cell viability

A cell viability assay was performed by using the LIVE/DEAD^®^ viability/cytotoxicity kit (Invitrogen) according to the manufacturer’s instructions. Briefly, the samples were loaded with polyanionic calcein-AM (4 μM, green fluorescence) and ethidium homodimer-1 (EthD-1, 4 μM, red fluorescence) for the detection of live and damaged cells, respectively, for 45 min. The percentage of dead cells was calculated by the EthD-1-positive total dead cells of Hoechst-positive cells.

### Electrical field stimulation

First, we labeled the fluorescence dye calcein-AM to easily track the biohybrid artificial muscle. The contraction of muscle fibers was stimulated by an electrical field of a 15-ms step pulse duration (80 V) at 0.5 Hz frequency under a fluorescence microscope (IX71, Olympus).

## Supplementary information

Supplementary Information

Supplementary movie 1
