# Supplementary material for: Biomimetic cell-actuated artificial muscle with nanofibrous bundles
Source: Microsyst Nanoeng. 2021 Sep 3;7:70. doi: 10.1038/s41378-021-00280-z (PMC8433352; doi:10.1038/s41378-021-00280-z)
Supplement: Supplementary file 1 — Supplementary Information [file 41378_2021_280_MOESM1_ESM.docx]

**Supplementary Information**

**Biomimetic cell-actuated artificial muscle with nanofibrous bundles**

Yongwoo Jang^1,^†, Sung Min Kim^2,^†, Eunyoung Kim^1^, Dong Yeop Lee^1^, Tong Mook Kang^3^, Seon Jeong Kim^1,^*

^1^Center for Self-Powered Actuation, Department of Biomedical Engineering, Hanyang University, Seoul 04763, South Korea

^2^Department of Physical Education and Human-Tech Convergence Program (BK21 Four), Hanyang University, Seoul 04763, South Korea

^3^Department of Physiology, Sungkyunkwan University School of Medicine, Suwon 16419, South Korea


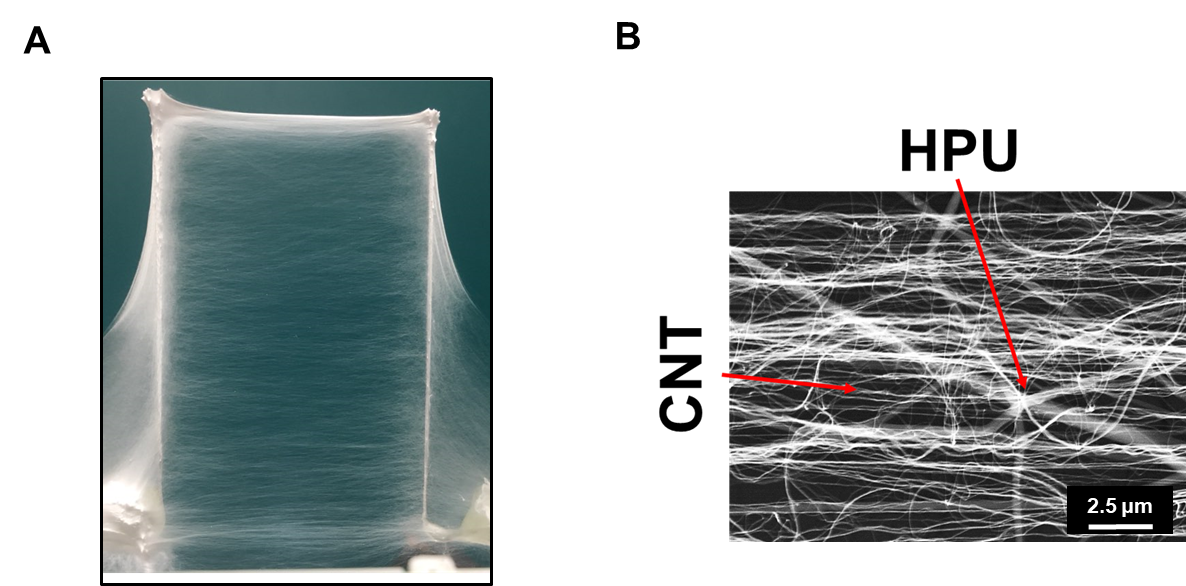


Figure S1. (A) Electrospun HPU fibers from a 5 wt% solution of HPU in 95% ethanol at an applied voltage of 15 kV. (B) SEM image of the HPU/CNT matrix.


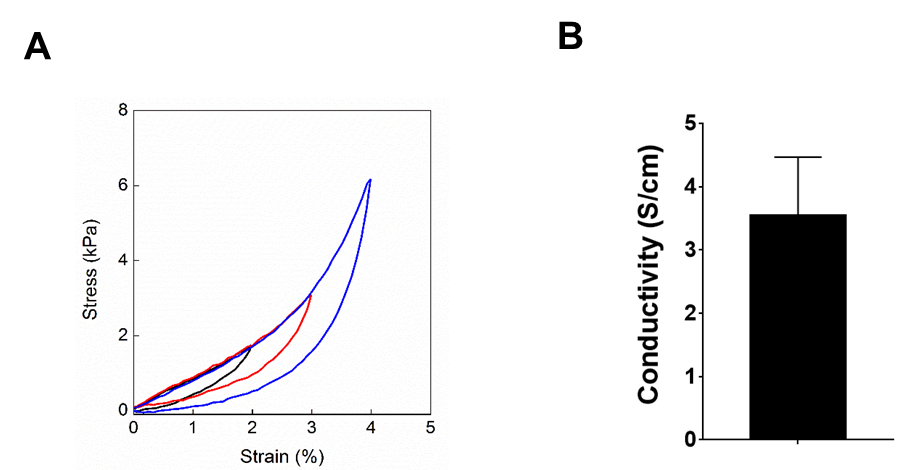


Figure Se. (A) Stress-strain curve of stretching and releasing for HPU/CNT fibers. (B) Electrical conductivity of HPU/CNT fiber.


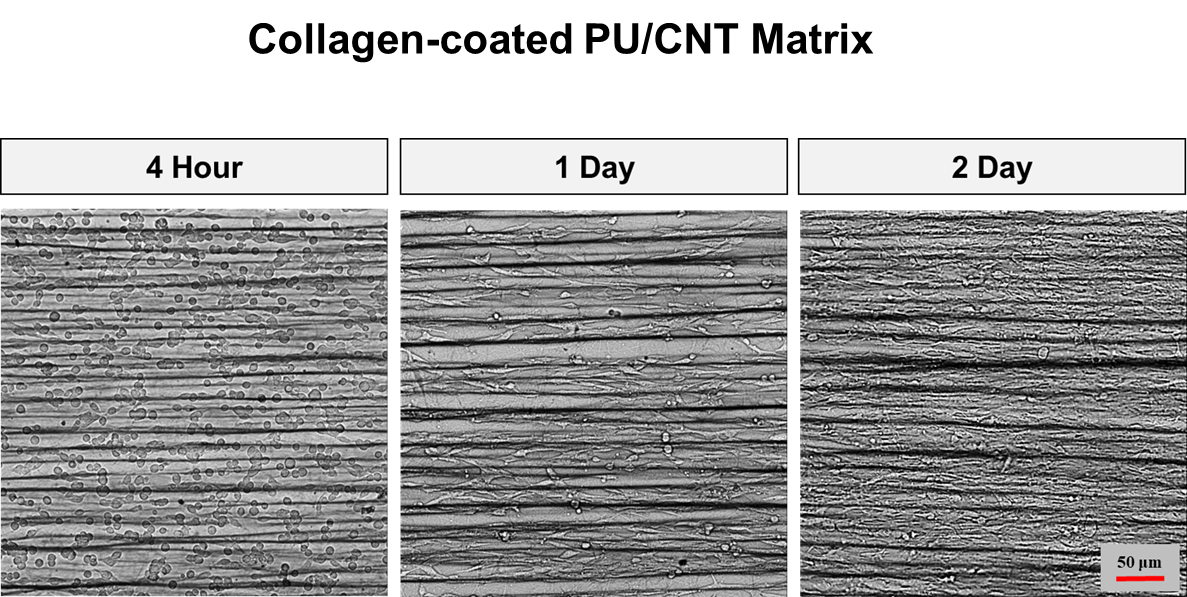


Figure S3. Proliferating C2C12 myoblasts on collagen-coated PU/CNT matrix for 2 days.


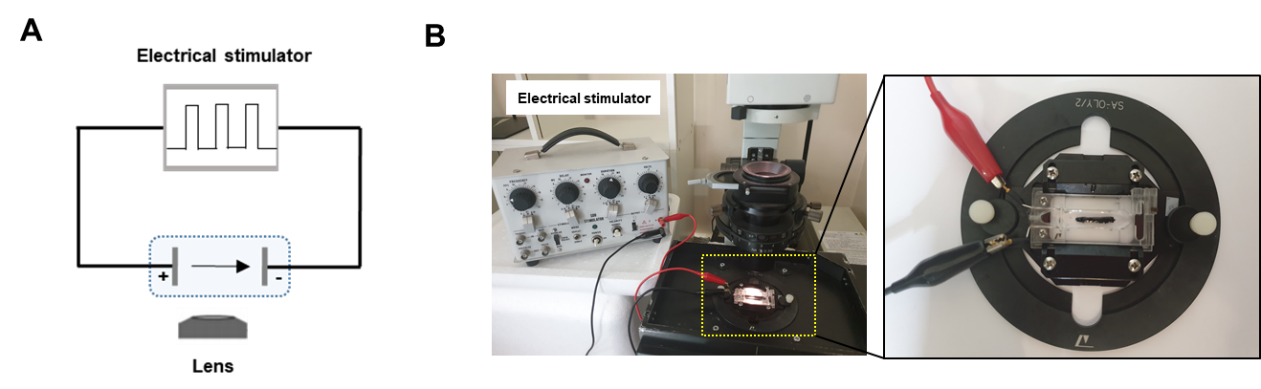


Figure S4. Schematic diagram and real picture of the device with a detection system and electric field stimulation.


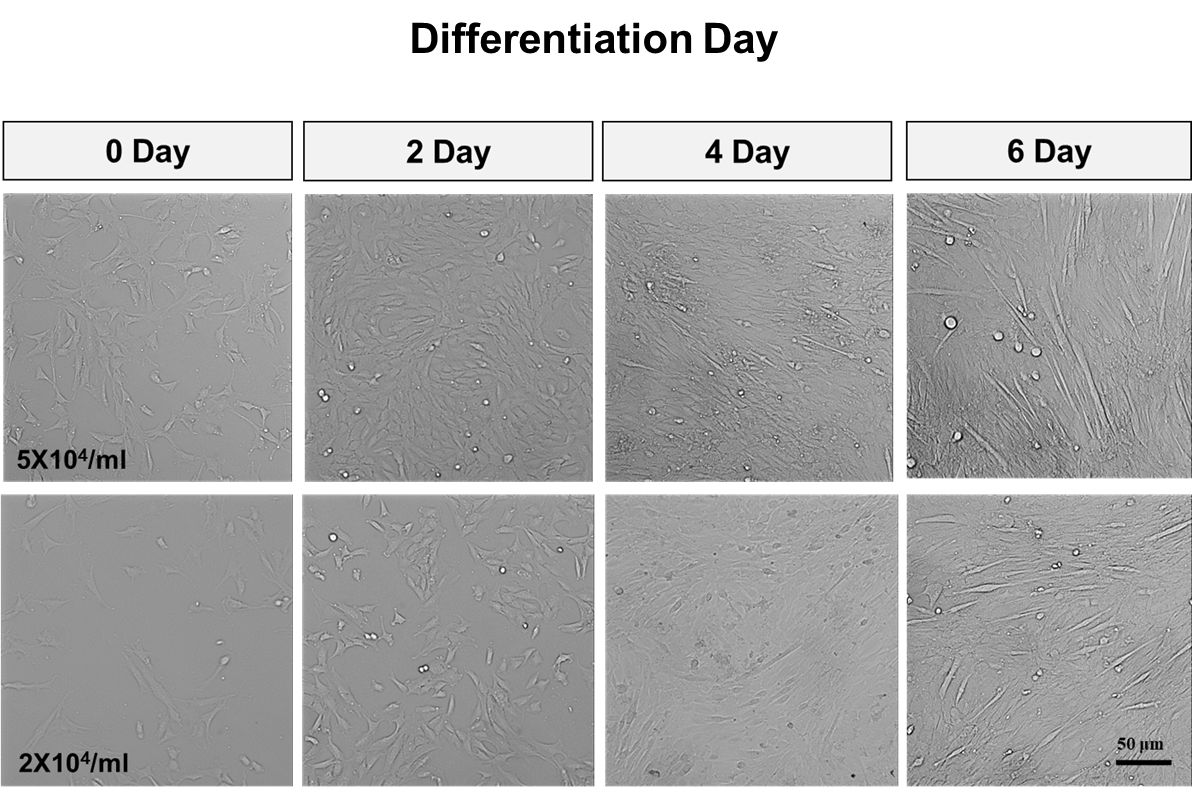


Figure S5. Culture conditions for the myogenic differentiation of C2C12 myoblasts on culture plates.


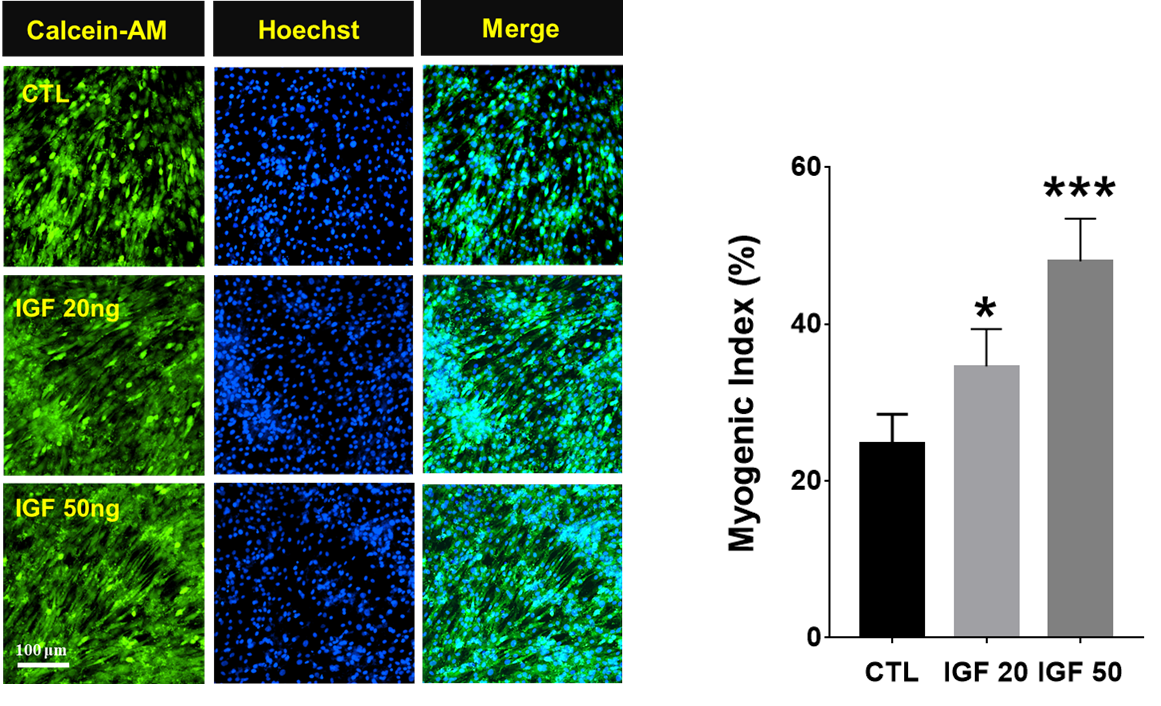


Figure S6. The supporting effect of IGF-1 supplementation on differentiated C2C12 cells on culture plates.
